# Human iPSC‐derived neural precursor cells differentiate into multiple cell types to delay disease progression following transplantation into YAC128 Huntington's disease mouse model

**DOI:** 10.1111/cpr.13082

**Published:** 2021-06-21

**Authors:** Hyun Jung Park, Juhyun Jeon, Jiwoo Choi, Ji Yeon Kim, Hyun Sook Kim, Ji Young Huh, Steven A. Goldman, Jihwan Song

**Affiliations:** ^1^ Department of Biomedical Science CHA Stem Cell Institute CHA University Seongnam‐si Korea; ^2^ Department of Neurology CHA Bundang Medical Center CHA University Seongnam‐si Korea; ^3^ Department of Laboratory Medicine CHA Bundang Medical Center CHA University Seongnam‐si Korea; ^4^ Center for Translational Neuromedicine University of Rochester Medical Center Rochester NY USA; ^5^ Center for Translational Neuromedicine University of Copenhagen Faculty of Health and Medical Science Copenhagen N Denmark; ^6^ iPS Bio, Inc. 3F, 16 Daewangpangyo‐ro 712 Beon‐gil Seongnam‐si Korea

**Keywords:** astrocyte, functional recovery, glutamate toxicity, human leukocyte antigen (HLA), Huntington’s disease (HD), induced pluripotent stem cell‐derived neural precursor cells (iPSC‐NPCs), inflammation

## Abstract

**Objectives:**

To investigate whether human HLA‐homozygous induced pluripotent stem cell (iPSC)‐derived neural precursor cells (iPSC‐NPCs) can provide functional benefits in Huntington’s disease (HD), we transplanted them into the YAC128 transgenic HD mouse model.

**Materials and Methods:**

CHAi001‐A, an HLA‐homozygous iPSC line (A*33:03‐B*44:03‐DRB1*13:02), was differentiated into neural precursor cells, and then, they were transplanted into 6 months‐old YAC128 mice. Various behavioural and histological analyses were performed for five months after transplantation.

**Results:**

Motor and cognitive functions were significantly improved in transplanted animals. Cells transplanted in the striatum showed multipotential differentiation. Five months after transplantation, the donor cells had differentiated into neurons, oligodendrocytes and astrocytes. Transplantation restored DARPP‐32 expression, synaptophysin density, myelin basic protein expression in the corpus callosum and astrocyte function.

**Conclusion:**

Altogether, these results strongly suggest that iPSC‐NPCs transplantation induces neuroprotection and functional recovery in a mouse model of HD and should be taken forward for clinical trials in HD patients.

## Graphical abstract

Human HLA‐homozygous induced pluripotent stem cell (iPSC)‐derived neural precursor cells (iPSC‐NPCs) have multilineage differentiation potential and provide neuroprotection and functional recovery when transplanted into the YAC128 transgenic mouse model of Huntington’s disease (HD).

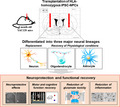

## 1 INTRODUCTION

Huntington’s disease (HD) is characterized by progressive motor, cognitive, and psychiatric disturbances associated with neuronal dysfunction and atrophy of the striatum and other brain regions.^1^ HD occurs when a mutant of the huntingtin protein with an expanded chain of poly‐glutamines in the N‐terminal region) accumulates and aggregates inside cells.^1^ Although clinical trials showed that transplantation of foetal neural progenitor cells was largely ineffective against HD,^2^ huntingtin lowering strategies (for instance with antisense oligonucleotides) showed promising results.^3^, ^4^ Conversely, human stem cell‐based neuro‐restorative or neurodegenerative strategies can offer an alternative therapeutic strategy. To date, human embryonic stem cell‐derived neural progenitor cells^5^ or mouse induced pluripotent stem cells (iPSC)‐derived neural stem cells^6^ have shown beneficial effects on HD transgenic mice. These studies primarily focused on the replacement of neurons such as medium spiny neurons (MSNs).

iPSC‐derived neural progenitor cells (iPSC‐NPCs) self‐renew and can differentiate into neurons as well as glia.^7^ After spinal cord injury in mice, transplanted human iPSC‐NPCs differentiated into the three major neural lineages (*ie*, neurons, astrocytes and oligodendrocytes), leading to functional recovery.

We previously reported that glial progenitor cell transplantation provided some benefits in HD mice.^8^ Engrafting normal human glial progenitor cells into the striatum of newborn R6/2 HD mice substantially replaced the diseased striatal glia with both human glial progenitor cells and their derived astrocytes, slowing the disease progression and increasing the survival of R6/2 mice.

In this study, we investigated the therapeutic efficacy of healthy cord blood‐derived human leukocyte antigen (HLA)‐homozygous iPSC‐NPCs on a YAC128 mouse HD model that exhibits striatal and cortical atrophy, as well as progressive deterioration of motor and cognitive functions.^9^, ^10^ We found that five months after transplantation, the grafted cells predominantly differentiated into DARPP‐32‐, O4‐ or GFAP‐positive cells, which had neuroprotective effects. Surprisingly, most of the therapeutic benefits came from astrocytes. Transplanted animals had higher striatum DARPP‐32 expression and improved motor and cognitive functions. Our results suggest that iPSC‐NPCs mainly differentiate into astrocytes, which delays neurodegeneration. Furthermore, given the benefits of using HLA‐homozygous iPSCs to treat HLA‐matched HD patients, our data strongly suggest that HLA‐homozygous iPSC‐derived NPCs have strong clinical merit and warrant future clinical trials.

## 2 MATERIALS AND METHODS

### 2.1 Lead Contact and materials availability

Further information and requests for resources and reagents should be directed to and will be fulfilled by the Lead Contact, Jihwan Song (jsong5873@gmail.com). This study did not generate new unique reagents.

### 2.2 Cell line

We used the HLA‐homozygous iPSC line, CHAi001‐A, because it carries the most frequent HLA‐homozygous haplotypes (A*33:03‐B*44:03‐DRB1*13:02). It was established from the frozen cord blood of a healthy donor using the episomal method.^11^ These haplotypes cover about 9% of the Korean population and are also relevant to other Asian populations (Japanese, Chinese, etc). This iPSC line can also treat diverse Asian populations in the United States, etc This study was previously approved by the Institutional Review Board of CHA University (1044308‐201511‐BR‐025‐01).

### 2.3 iPSC culture and differentiation into NPCs

CHAi001‐A cells were maintained in StemFit Basic02 medium (Ajinomoto, Japan) supplemented with 100 ng/ml basic fibroblast growth factor (bFGF, Peprotech) and 10 umol/L Y27632 (ROCK inhibitor, Peprotech) for about seven days before they were treated with a TrypLE solution (GIBCO) for 5 minutes at 37°C in a CO_2_ incubator. The dissociated cells were cultured in the neural differentiation medium that consists of DMEM/F12 (Invitrogen) supplemented with 1% antimycotic‐antibiotics, 1% nonessential amino acids (NEAA, GIBCO), 0.1% beta‐MeOH, 20% Knockout^TM^ serum replacement, 10 umol/L SB431542 (Reagent Direct), 100 nmol/L LDN193189 (Sigma) and 30 ng/ml ROCK inhibitor (Y‐27632, PeproTech) at 37°C in a CO_2_ incubator for neural induction. The cells were maintained in the neural differentiation medium for eight days. Embryoid bodies were dissociated in the NPC medium (DMEM/F12 supplemented with 1:100 antimycotic‐antibiotics (Welgene), 1:100 NEAA, sodium pyruvate (GIBCO), D‐glucose (Sigma Aldrich), L‐glutamine (Welgene), 1:1000 beta‐MeOH, 1:50 B‐27 (without vitamin A, GIBCO) and 20 ng/ml bFGF) in a dish coated with poly‐L‐ornithine (Sigma Aldrich) and laminin (Sigma Aldrich). Cells were split with Accutase (Stem Cell Technologies).

### 2.4 Flow cytometry analysis

When iPSCs reached ~80% confluency, we conducted fluorescence‐activated cell sorting (FACS). The cultures were dissociated into a single‐cell suspension using 0.5× TrypLE solution comprised of TrypLE Select Enzymes (Thermo Fisher Scientific) and 0.5 mmol/L UltraPure EDTA (Thermo Fisher Scientific). Cells remained unfixed and were stained with PE‐conjugated antigen‐specific antibodies and corresponding isotypes using the manufacturer's recommended concentration. We used anti‐SSEA4 (1:200) and anti‐IgM isotype (1:200), anti‐TRA‐1‐60 (1:200), anti‐TRA‐1‐81 (1:200) and anti‐IgG3 isotype (1:200; all from BD Biosciences). After staining, the required amounts of samples were processed by a BD FACSCalibur (BD Biosciences). Data were obtained and analysed using BD FACS Diva software.

### 2.5 Reverse transcription‐polymerase chain reaction (RT‐PCR)

Total RNAs were isolated using the TRIzol reagent (Thermo Fisher Scientific, 15596026). Complementary DNAs (cDNAs) were synthesized using a cDNA synthesis Kit (CMRTK002; Cosmo Genetech). RT‐PCR amplification was conducted in a final volume of 20 μl containing 200 ng/μl cDNA for each sample. The following primers were used: GAPDH (forward primer: TGA CCA CAG TCC ATG CCA TCA CTG C; reverse primer: GTC ATA CCA GGA AAT GAG CTT GAC A); Oct3/4 (forward primer: CTG AAG CAG AAG AGG ATC AC; reverse primer: GAC CAC ATC CTT CTC GAG CC); Nanog (forward primer: TTC TTG ACT GGG ACC TTG TC; reverse primer: GCT TGC CTT GCT TTG AAG CA); Sox2 (forward primer: GCT GCA AAA GAG AAC ACC AA; reverse primer: CTT CCT GCA AAG CTC CTA CC); Lin28 (forward primer: CAC CAT GGG CTC CGT GTC CAA CCA GCA G; reverse primer: TCA ATT CTG TGC CTC CGG GAG CAG GGT AGG); Nestin (forward primer: TCC AGA AAC TCA AGC ACC A; reverse primer: AAA TTC TCC AGG TTC CAT GC); Sox2 (forward primer: GCT GCA AAA GAG AAC ACC AA; reverse primer: CTT CCT GCA AAG CTC CTA CC); and Musashi (forward primer: ACA GCC CAA GAT GGT GAC TC; reverse primer: CCA CGA TGT CCT CAC TCT CA).

### 2.6 Quantification of differentiated cells in vitro

We measured the number of the differentiated cells NeuN, GFAP‐ and O4‐positive cells on plate using the IXMC (ImageXpress Micro Confocal) high‐content imaging system (Molecular Devices). The differentiated cells on plate were delineated at 20X magnification (n = 3). The merged positive cells were counted by the scoring module of MetaXpress software (Molecular Devices).

### 2.7 YAC128 transgenic mice and transplantation

All the experiments were performed on YAC128 transgenic and wild‐type (FVB/N) mice maintained on the FVB/N strain background.^12^ Mice were bred in the animal facility of CHA Bio Complex (Pangyo, Korea). The Institutional Animal Care and Use Committee (IACUC 200019) of CHA University approved all experiments. YAC128 mice exhibit glutamate toxicity from three months of age ^13^, ^14^ and motor function defects from six months of age.^9^ In this study, we used six‐month‐old YAC128 mice to investigate whether HLA‐iPSC‐NPCs transplantation improved motor function deficits. In the transplanted group (TP, n = 10), we stereotaxically injected 4 μl of HLA‐iPSC‐NPCs (100 000 cells/μl) into the striatum of both hemispheres using the following coordinates: AP + 0.5 mm, ML ± 1.8 mm, and DV −3 and −4 mm from the bregma. In the sham group (n = 10), we injected 4 μl of suspension medium (DMEM) parallelly into both hemispheres. In both groups, we intraperitoneally injected cyclosporine A (5 mg/kg, Sigma) three days before the transplantation and daily for months (until sacrifice).

### 2.8 Behavioural tests

We performed motor function tests (rotarod and grip strength tests) and cognitive function tests (simple swim and novel object recognition tests) for five months to evaluate the effect of cell transplantation in six‐month‐old YAC128 transgenic mice. To investigate the cell survival and the changes of pathology development after transplantation, two or three mice from each group were sacrificed for histological analysis at different time points of the behavioural analysis (Wild type (WT): n = 8, Sham: n = 7, and TP: n = 8).

### 2.9 Motor function tests

#### 2.9.1 Accelerating rotarod test

We used an accelerating rotarod protocol (San Diego Instruments) to test motor coordination and gait changes. Accelerations ranged from 0 to 45 rpm over a period of 2 minutes. After the training period (two trials per day for three days), mice were tested for three consecutive trials in a single day and allowed 1.5 hours of rest time between the trials. The rotarod was wiped clean with ethanol between each subject and trial.

#### 2.9.2 Grip strength test

To further assess motor function, we performed a grip strength test.^15^ The apparatus (San Diego Instruments) comprised an adjustable grip (6 cm wide, 0‐45) connected to a digital gauge. For this measure, the mice were lifted by the tail so that their forepaws grasped the grip. The mice were then gently pulled backward by the tail until they released the wire. The maximal force exerted before the mice lost their grip was recorded. Each mouse was tested nine times, and the average of the three highest scores was used for subsequent analyses.

### 2.10 Cognitive function tests

#### 2.10.1 Simple swim test

We chose the simple swim test to measure cognitive impairments and procedural learning, as previously performed in HD animals.^9^, ^16^ Mice were placed in the centre of a linear swimming chamber (76 × 13 cm; water depth: 9 cm; platform: 6 × 13 cm) facing away from the escape platform. The amount of time required to reach the platform and the initial swimming direction were recorded for each trial. Swimming towards the platform was arbitrarily given a score of 0, whereas swimming away from the platform was given a score of 1. The mice were trained for three days in three pairs of two consecutive trials with an interval of 2 hours between each trial. For each mouse, the average of the three trials on the last day of testing was used for analysis.^16^

#### 2.10.2 Novel object recognition test

The novel object recognition test measures rodent recognition memory.^17^ The experimental apparatus consisted of a white rectangular open field (45 cm × 45 cm × 45 cm). Before training, mice explored the open field for 10 minutes and then rested for 5 minutes in the cage. Two identical objects were placed at 7 cm from both upper corners of the chamber, and the mice were placed in the lower‐left corner of the chamber. They were then allowed to explore the field for 7 minutes. The mouse was then removed from the chamber and placed in the cage for 5 minutes. To evaluate the mouse's reaction to a new object, the upper‐right corner object was replaced with an unfamiliar new object and the experimental animal was again placed in the lower‐left corner for 5 minutes. The test was performed three and five months after transplantation. Exploratory activity in the experimental arena was measured using EthoVision XT 11.5 (Noldus).

#### 2.10.3 Western blot analysis

Brain tissues were lysed with RIPA buffer (150 mmol/L NaCl, 1% Nonidet P‐40, 0.5% deoxycholic acid, 0.1% SDS and 50 mmol/L Tris‐HCl, pH 7.4) containing a protease inhibitor (Roche), and then sonicated for 1 minutes and incubated on ice for 20 minutes. The supernatant was collected and centrifugated at 13,200 *g* for 15 minutes at 4°C, and protein concentrations were determined using a BCA assay (Thermo Fisher Scientific). Equal amounts of proteins were then loaded on 8%‐12% SDS polyacrylamide gels. The separated samples were transferred to a PVDF membrane and incubated with the primary antibodies against the target proteins and then with the HRP‐conjugated secondary antibodies. The protein bands were visualized by enhanced chemiluminescence (ECL; Millipore) using a bioimaging analyser (Bio‐Rad). The relative intensity of each band was measured using Image J software (rsb.info.nih.gov, by W. Rasband). Primary antibodies used: human GFAP (R&D Systems, 1:1000), EAAT2 (Santa Cruz, 1:1000), Kir4.1 (Millipore, 1:1000), Arginase‐1 (Santa Cruz, 1:1000) and IL‐1β (Santa Cruz, 1:1000).

#### 2.10.4 Immunocytochemistry and Immunohistochemistry

Cells were fixed using a 4% paraformaldehyde solution for iPSC characterization or 42 days after neural differentiation. YAC128 mice used in this experiment were sacrificed five months after cell transplantation when all the behavioural analyses were completed. All mice were perfused with 5 μl/ml heparin and then with a 4% paraformaldehyde solution. Mouse brains were fixed in the 4% paraformaldehyde solution overnight and left to soak in 30% sucrose solution for about three days. The brain tissues were then frozen using an optimal cutting temperature compound and sectioned into 30 µm thick slices using a cryotome (CM3050, Leica, Germany). The cryosectioned brain tissues were stored in a cryoprotective solution (40% glycerol, 40% ethylene glycol and 20% 0.2 M phosphate buffer solution) at −20°C. The fixed cells or brain slices were incubated in a blocking buffer (10% donkey serum and 0.3% Triton X‐100 in phosphate‐buffered solution) for 30 minutes at room temperature. The samples were then incubated with primary antibodies overnight at 4°C and then with the appropriate fluorescent probe‐conjugated secondary antibodies for 1 h at room temperature protected from light. Nuclei were stained with 4,6‐diamidino‐2‐phenylindole (DAPI, Thermo Fisher Scientific) at a 1:5,000 dilution. Images were captured by a confocal microscope (LSM880, Zeiss). For image analysis of pixel numbers, we used the co‐localization module of Zen black software of LSM9 confocal microscope (Zeiss). Specific primary antibodies used: OCT4, Nanog, SSEA4, Tra‐1‐81, SMA, AFP, NESTIN, SOX2, MUSASHI, Tuj1, S100β, human GFAP (R&D systems, 1:1000), human NESTIN, human MAP2, synaptophysin, EAAT2 (Santa Cruz, 1:1000), Kir4.1 (Santa Cruz, 1:1000), Arginase‐1(Santa Cruz, 1:1000), IL‐1β (Santa Cruz, 1:1000), hDARPP‐32 (Cell signaling, 1:200), Ctip2 (Abcam, 1:200), GFAP (BD and DAKO, 1:200), Iba‐1 (Wako, 1:200), O4 and myelin basic protein (MBP) (Millipore, 1:100).

#### 2.10.5 **Quantification of differentiated cells** in vivo

We measured the number of the merged MAP2‐, GFAP‐, O4‐ and hNu‐double‐positive cells in the striatum (AP 0 mm, 0.5 mm; TP site, 1.0 mm) using the ImageXpress Micro Confocal high‐content imaging system (Molecular Devices). The striatum was delineated at 20 × magnification (n = 3). The merged positive cells were counted in all regions of the three sections and by the scoring module of MetaXpress software (Molecular Devices).

#### 2.10.6 Quantification of the striatal density

We measured the striatal density of DARPP‐32 (Millipore, 1:200)‐positive areas by immunostaining to investigate the neuroprotective effects of the transplanted cells. The DARPP‐32 immunostaining in the striatum was estimated using an optical fractionator and unbiased stereology of stained cells. To measure striatal density, three images of DARPP‐32 immunostaining near the middle of the striatum were captured. The images were delineated at 4× magnification, and then, the density of images was quantitated using Image J software (rsb.info.nih.gov, by W. Rasband).

#### 2.10.7 Morphology analysis

We measured the number of processes, the maximal process length, the outgrowth intensity, and the body area of GFAP‐ or Iba‐1‐positive cells in striatal regions from three or six sections from three brains in each experimental group. We obtained images using a Nikon microscope or ImageXpress Micro Confocal high‐content imaging system (Molecular Devices) and connected to the stage and the computer with the distance information in the z‐axis. Each region was analysed using the outgrowth module of MetaXpress software (Molecular Devices).

#### 2.10.8 Cell counting

The unbiased stereological estimation of the total number of human Nuclei (hNu)‐, DARPP‐32‐ and Iba‐1‐positive cells, and the merged GFAP‐ and hNu‐double‐positive cells in the striatum was performed using the ImageXpress Micro Confocal high‐content imaging system (Molecular Devices). The sections used for counting covered the entire striatum (2 mm^2^). This generally yielded sections in a series. The positive cells were counted in all regions of the six to seven sections. Sampling was performed using the ImageXpress Micro Confocal high‐content imaging system (Molecular Devices), which was connected to the stage and fed the computer with the distance information in the z‐axis. The striatum was delineated at 20 × magnification. We excluded the guard volume (4 μm from the top and 4‐6 μm from the bottom of the section) from both surfaces to avoid the problem of the lost cap, and only the profiles that came into focus within the counting volume (with a depth of 10 μm) were counted by the scoring module of MetaXpress software (Molecular Devices). The total number of cells was calculated according to the optical fractionator formula.^18^

#### 2.10.9 Statistical analysis

Data were analysed with two‐way ANOVA followed by Tukey's post hoc test or Student's *t* test, using SPSS software (version 10.0; Chicago, IL) or GraphPad Prism (version 5.0; San Diego). Significance was accepted at the 95% probability level. Data are presented as mean ± SEM.

## 3 RESULTS

### 3.1 Characterization and differentiation of HLA‐homozygous iPSC

We assessed the effect of iPSC‐NPCs transplantation on an HD animal model. We previously established ten most frequent HLA‐homozygous iPSCs in the Korean population. They can be transplanted in 41.07% of the Korean population with minimal immune suppression.^11^, ^19^

**FIGURE 1:**
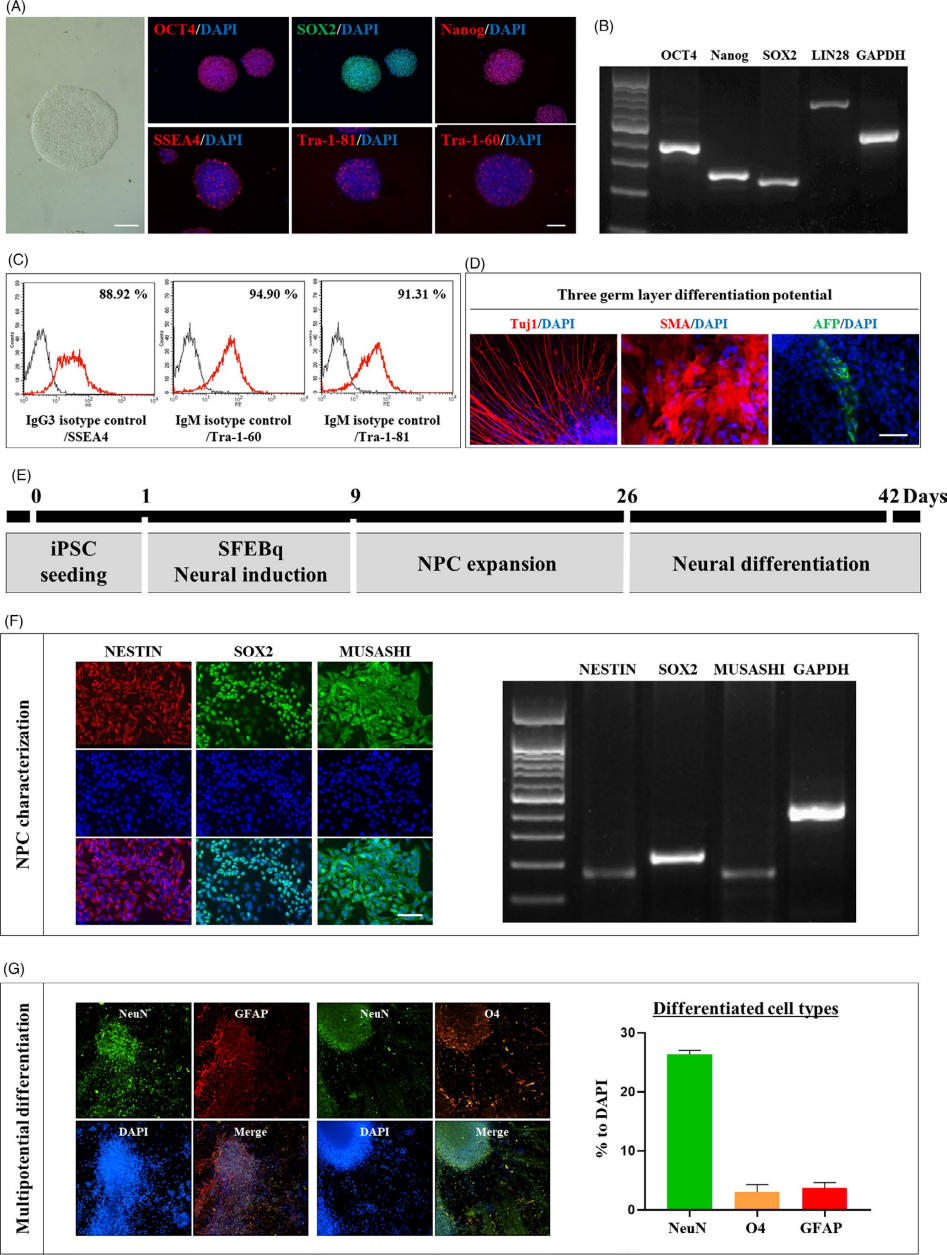
Characterization and differentiation of HLA‐homozygous iPSC. (A) Immunostaining for OCT4, SOX2, Nanog, SSEA4, Tra‐1‐81 and Tra‐1‐60 showing the characterization of HLA‐iPSC. Scale bar: 100 μm. (B) HLA‐iPSC characterization by RT‐PCR. (C) HLA‐iPSC characterization by FACS analysis. (D) Immunostaining for Tuj1, SMA and AFP showing differentiation potential into the three germs layers. Scale bar: 50 μm. (E) Neuronal precursor cells (NPCs) generation method. (F) Immunostaining and RT‐PCR analyses for NESTIN, SOX2 and MUSASHI showing the characterization of the HLA‐iPSC‐NPCs. Scale bar: 50 μm. (G) Six weeks after differentiation, the ratio of differentiated cells was measured using IXMC analysis showing that HLA‐iPSC‐NPCs expressed markers for neurons (NeuN; 26.3 ± 0.69%), oligodendrocytes (O4; 3.0 ± 1.21%) and astrocytes (GFAP; 3.7 ± 0.94). Scale bar: 100 μm.

First, we characterized iPSCs for transplantation. Immunocytochemical analysis showed that the iPSC colonies expressed high levels of pluripotent markers such as OCT4, SOX2, NANOG, SSEA4, TRA‐1‐81 and TRA‐1‐60 (Figure 1A). Semi‐quantitative RT‐PCR analysis showed high expression levels of Oct4, Sox2, Nanog and Lin28 (Figure 1B). Flow cytometry analysis showed a strong expression of cell surface antigens, such as SSEA4, TRA‐1‐60 and TRA‐1‐81 (Figure 1C). The iPSCs differentiated into three germ layers, as the formation of neural rosettes (ectoderm, Tuj‐1 staining), cartilage (mesoderm, SMA staining) and gut epithelium (endoderm, AFP staining; Figure 1D) indicated.

Next, we applied the embryoid body‐based neural differentiation method to differentiate these iPSCs into NPCs (Figure 1E). Immunocytochemical and RT‐PCR analyses indicated that iPSC‐NPCs expressed NESTIN, SOX2 and MUSHAHI, confirming their identities (Figure 1F). We then examined whether HLA‐iPSC‐NPCs could differentiate into neurons, oligodendrocytes or astrocytes (Figure 1G). Six weeks after differentiation, IXMC‐based molecular imaging analysis revealed that HLA‐iPSC‐NPCs expressed markers for neurons (NeuN; 26.3 ± 0.69%), oligodendrocytes (O4; 3.0 ± 1.21%) and astrocytes (GFAP; 3.7 ± 0.94) (Figure 1G). Thus, HLA‐iPSC‐NPCs are multipotent neural progenitors capable of differentiating into the three major neural lineages (neurons, oligodendrocytes and astrocytes), although the majority of cells consisted of neurons under this differentiation condition.

### 3.2 HLA‐iPSC‐NPCs improved motor and cognitive function

**FIGURE 2:**
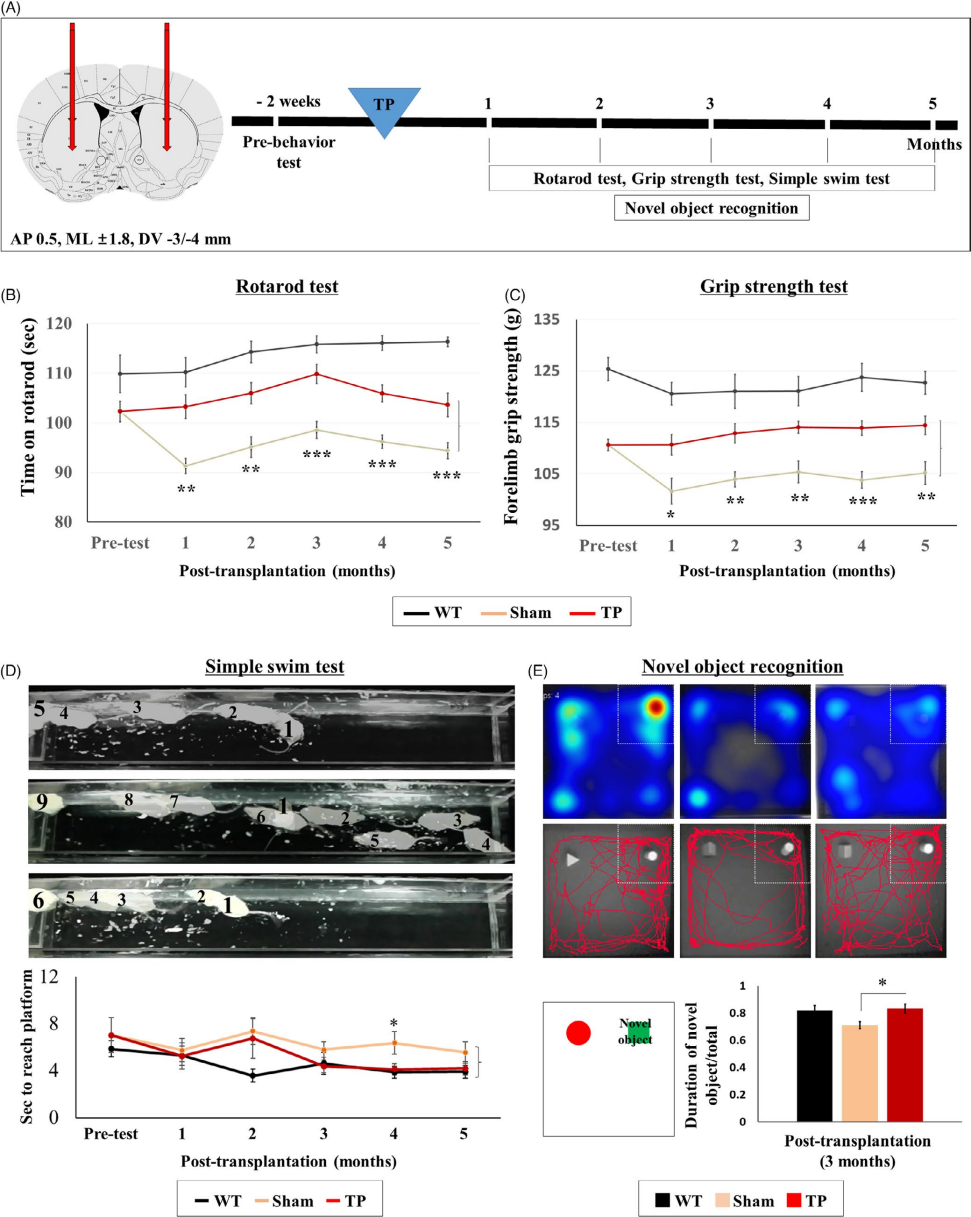
HLA‐iPSC‐NPCs transplantation leads to functional recovery in 11 months‐ol YAC128 transgenic mice. (A) Scheme of the in vivo experiment. Wild‐type (WT) mice were injected with media alone, whereas six‐month‐old YAC128 mice were transplanted with HLA‐iPSC‐NPCs. Behavioural tests were performed every month for six months after transplantation. (B, C) Motor function recovery was monitored every month using the accelerating rotarod and grip strength tests (WT: n = 8, Sham: n = 7, and TP: n = 8, *P <.05, **P <.01 and ***P <.001). (D, E) Cognitive recovery was monitored using the simple swim and novel object recognition tests (WT: n = 8, Sham: n = 7, and TP: n = 8). (D) Simple swim test results. (E) Novel object recognition test results. Data were analysed by two‐way ANOVA followed by a Tukey post hoc test, using the SPSS software

To assess the efficacy of HLA‐iPSC‐NPCs against HD, we transplanted them into YAC128 transgenic mice. Figure 2A shows the experimental design. Five months after transplantation, we observed many human nuclei (hNu)‐positive cells in the striatum (Figure S1A), suggesting that the donor HLA‐iPSC‐NPCs survived. The striata of transplanted mice had approximately 135,120 ± 312 hNu‐positive cells, corresponding to approximately 33.8% of the total number of transplanted cells (4 × 10^5^) (Figure S1B). To quantify the contributions of transplanted human iPSC‐derived neural precursor cells, we selected three striatal tissues from three transplanted animals. As shown in Figure S3, the percentages of double‐positive cells against human nuclei antigen (hNu) in each analysis were as follows: MAP2+/hNu+ (25.45 ± 6.4%), O4+/hNu+ (2.85 ± 1.5%) and GFAP+/hNu+ (66.62 ± 8.2%). The total average of proportions of each cell type in the transplants when adjusted to hNu + cells were as follows: MAP2+ neurons (26.04%), O4+ oligodendrocytes (2.6%) and GFAP + astrocytes (66.03%). Based on this analysis, it appears that astrocytes would play an important role in ameliorating the toxic environment in the HD brains, which were indeed supported by histological analyses (Figure 6). The remaining percentage of different cell types (~4.73%) has yet to be identified.

We examined whether the transplanted HLA‐iPSC‐NPCs rescued motor function. We performed the rotarod test and the grip strength test every month for five months after transplantation. The TP group showed significantly better performance than the sham group in both the rotarod test (Figure 2B, *P* < .001; see also Video S1) and the grip strength test (Figure 2C, *P* < .01; see also Video S2). Interestingly, we observed a decline pattern after three months in the case of rotarod test, but further analysis revealed that no statistical differences were observed after three months (*ie*, 3 months vs. 4 months; 4 months vs. 5 month; and 3 months vs. 5 months) (Figure S2).

We also assessed cognitive function by performing the simple swim test and the novel object recognition test. After transplantation, we performed the simple swim test every month for five months. Mice from the sham group reached the visible escape platform twice slower than WT mice. They also had aberrant swimming direction patterns, reminiscent of motor coordination and navigational memory impairments (Figure 2D, Sham and TP: *P* < .05; see also Video S3). Interestingly, three months after transplantation, the motor coordination and navigational memory of the TG group continually improved compared to the sham group. Besides, four months after transplantation, the TP group reached the platform significantly faster than it previously did (Figure 2D, *P* < .05; see also Video S3). We also performed the novel object recognition test three months after transplantation to measure short‐term memory in a non‐stressful experimental setting. As the heat map shows, the TP group explored the novel object significantly longer than the sham group. ANOVA analysis further demonstrated that transplantation significantly increased the novel object exploration time (Figure 2E, *P* < .05; see also Video S4). Altogether, these results strongly suggest that HLA‐iPSC‐NPCs transplantation improved motor and cognitive functions in YAC128 TG mice.

### 3.3 Transplanted HLA‐iPSC‐NPCs differentiated into neurons in YAC128 TG mice

We examined whether HLA‐iPSC‐NPCs transplanted in the striatum differentiated into the three major neural lineages (neurons, oligodendrocytes and astrocytes), as NPCs do.^20^

**FIGURE 3:**
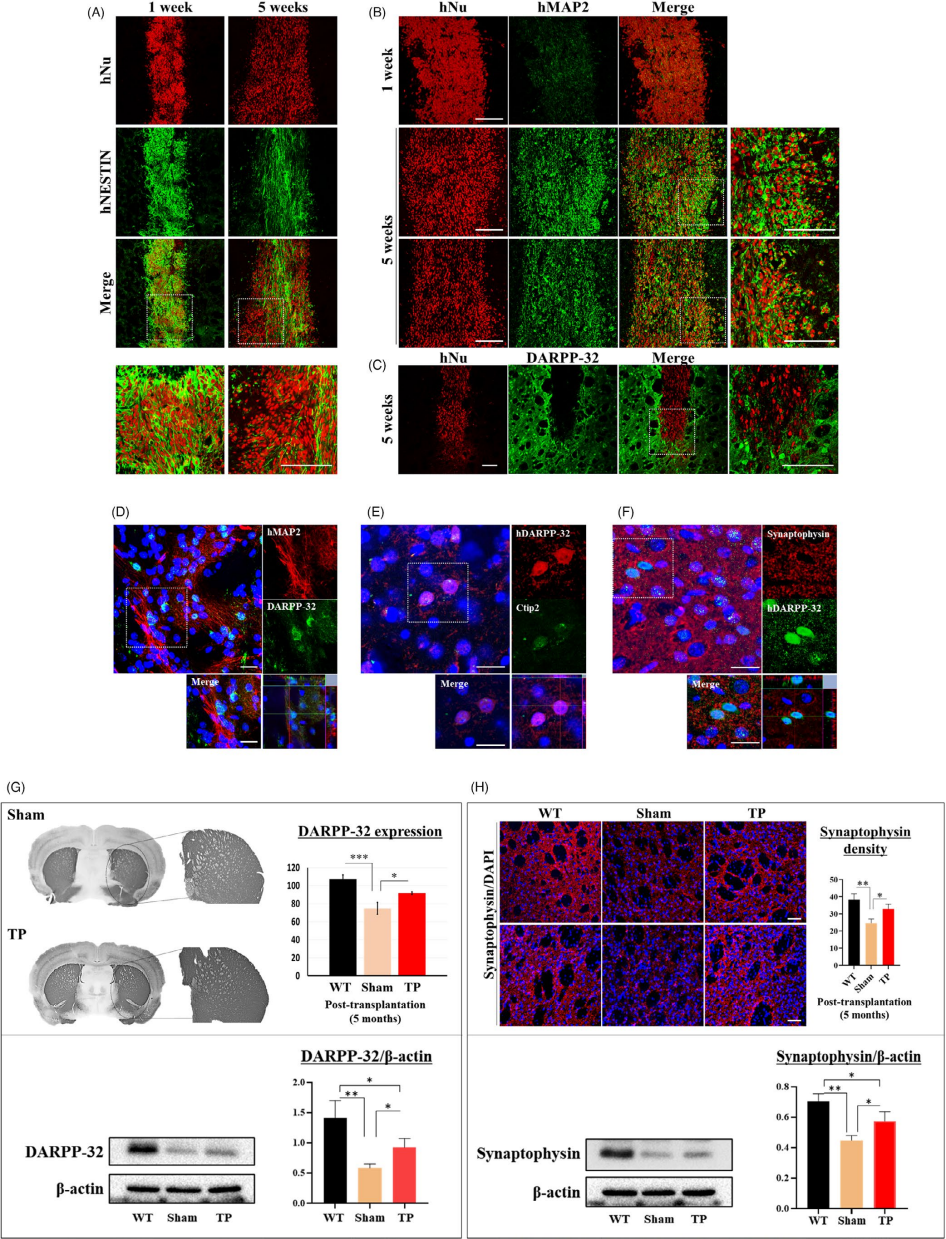
Transplanted iPSC‐NPCs contribute to the differentiation of specific neurons and confer neuroprotective effects in YAC128 transgenic mice. (A) Double staining for the neural precursor cell marker hNESTIN and hNu at one and five weeks (n = 1 each). Scale bar: 50 μm. (B) Double staining of the mature neuron marker hMAP2 and hNu at one and five weeks (n = 1 each). Scale bar: 50 μm. (C, D) Double staining for the medium spiny neuron (MSN) marker DARPP‐32 or hDARPP‐32 and hNu or hMAP2 at five weeks (n = 1) and five months (n = 3). Scale bar: 50 μm. n = 1. (D) Double staining showing the formation of DARPP‐32‐positive MSNs at five months after transplantation (n = 3). Scale bar: 100 μm. (E) Double staining for the MSN markers hDARPP‐32 and Ctip2 at five months (n = 3). Scale bar: 100 μm. (F) Double staining for the synaptic marker synaptophysin and hDARPP‐32 showing the possibility of synapse formation from the differentiated MSNs at five months (n = 3). Scale bar: 100 μm. (G) Immunostaining and Western blot analyses for DARPP‐32 expression in the striatum. Quantification of DARPP‐32 expression using Image J analysis (n = 3 in each group, *P < .05, ***P < .001). (H) Immunostaining and Western blot analyses for synaptophysin‐positive area in the striatum. Quantification of synaptophysin‐positive area using Image J analysis (Scale bar: 50 μm, n = 3 in each group, *P < .05, **P < .01). Data were analysed by two‐way ANOVA followed by a Tukey post hoc test using the SPSS software

First, we double‐stained hNu‐positive cells with antibodies against human‐specific NESTIN (hNESTIN) or MAP2 (hMAP2) at one and five weeks after transplantation (Figure 3A,B). One week after transplantation, hNu‐ and hNESTIN‐positive cells had merged and converged near the transplantation site. However, five weeks after transplantation, hNu‐ and hNESTIN‐positive cells merged less, and hNu‐positive cells diffused in the transplantation site (Figure 3A). In contrast, hNu‐ and hMAP2‐positive cells merged more at week five than at week one, and diffused hNu‐positive cells mainly merged with hMAP2‐positive cells (Figure 3B).

To observe the differentiation of specific striatal neurons, we double‐stained cells with antibodies against hNu or hMAP2 and DARPP‐32 or hDARPP‐32 (Figure 3C,D) five weeks and five months after transplantation. Five weeks after transplantation, hNu‐ and hMAP‐positive cells had merged, but hNu‐ and DARPP‐32‐positive cells had not (Figure 3C). However, five months after transplantation, we detected merged hMAP‐ and hDARPP‐32‐positive cells (Figure 3D).

We observed that transplanted cells formed DARPP‐32‐positive medium spiny neurons (MSNs). We further investigated this using Ctip2, which controls the differentiation of MSNs. In the absence of Ctip2, MSNs do not fully differentiate,^21^ making it a useful marker of striatal MSN formation. Interestingly, we found that the hDARPP‐23‐positive cells were Ctip2‐positive (Figure 3E). We also observed that hDAPP‐32‐positive cells expressed synaptophysin, suggesting the possibility of synapse formation from the transplanted cells (Figure 3F).

Next, we investigated whether transplanted HLA‐iPSC‐NPCs protect against HD. We quantified the striatal density of DARPP‐32‐ or synaptophysin‐positive areas in three brain sections of three mice from each group using Image J software. Interestingly, the TP group had significantly higher DARPP‐32 and synaptophysin expression levels (Figure 3G,H). Western blot analysis further confirmed the increase in DARPP‐32 and synaptophysin expression levels. These results strongly suggest that HLA‐iPSC‐NPCs transplantation preserved neurons.

### 3.4 Transplanted HLA‐iPSC‐NPCs differentiated into oligodendrocytes in YAC128 TG mice

**FIGURE 4:**
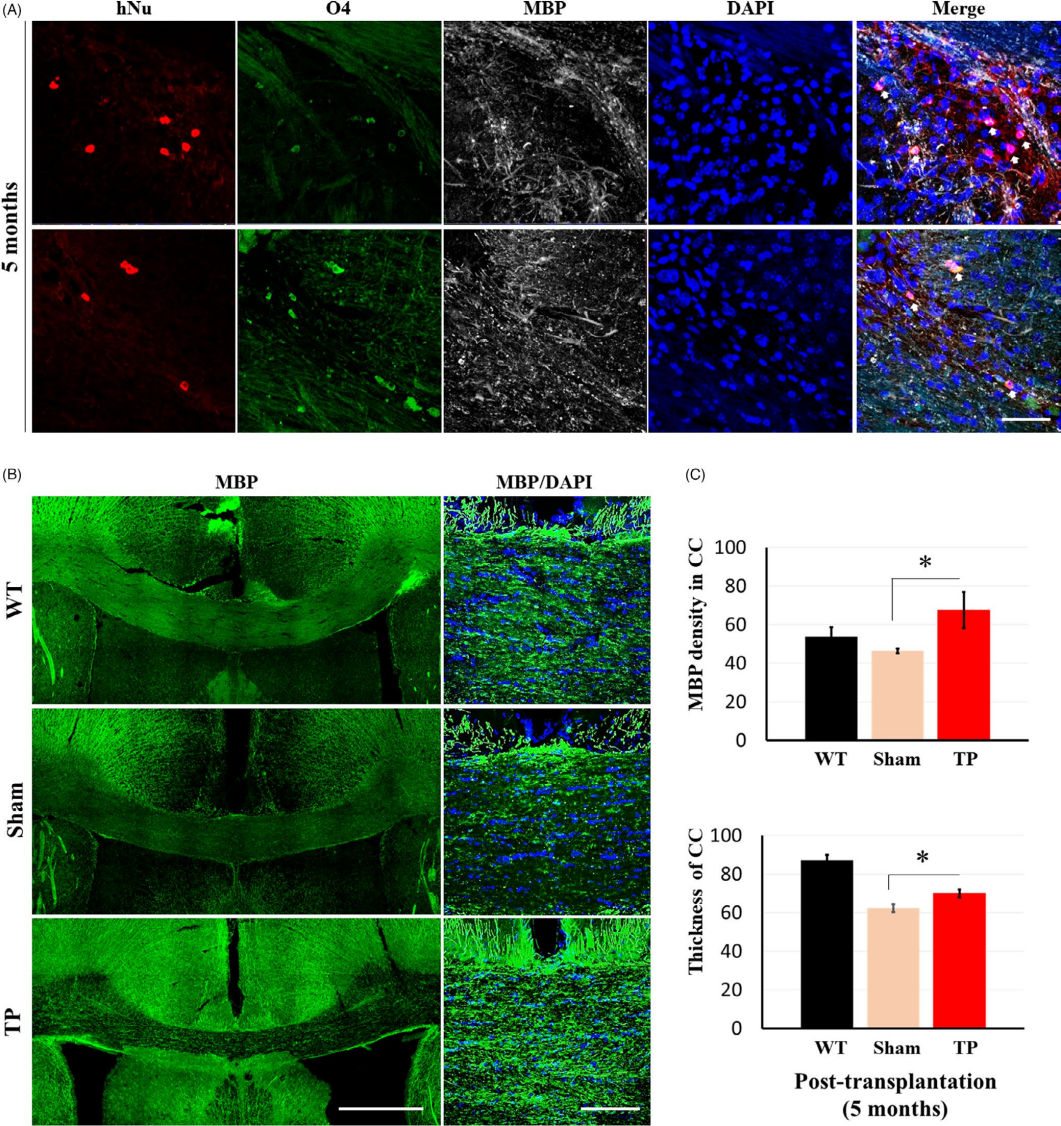
Transplanted HLA‐iPSC‐NPCs contribute to the differentiation and increase myelination of corpus callosum (CC) in 11‐month‐old YAC128 transgenic mice. (A) Triple staining for the oligodendrocyte markers O4 and MBP, and hNu (Scale bar: 50 μm. White arrow: triple merged cells). (B) Immunostaining for the identification of myelination exhibited MBP‐positive area in CC (scale bar: 500 μm or 100 μm). (C) Quantification of MBP density and thickness of CC (n = 3 in each group, *P < .05). Data were analysed by two‐way ANOVA followed by a Tukey post hoc test using the SPSS software

Next, we examined whether HLA‐iPSC‐NPCs differentiated into oligodendrocytes in the striatum. Five months after transplantation, we triple‐stained hNu‐positive cells with antibodies against O4 and MBP (Figure 4A). As Figure 4A shows, we found hNu‐, O4 and MBP‐positive cells near the corpus callosum (CC). Besides, the immunohistochemical analysis using an antibody against MBP showed that transplantation significantly increased the density and thickness of the CC (Figure 4B,C). Altogether, these results strongly suggest that HLA‐iPSC‐NPCs transplantation preserved myelin.

### 3.5 Transplanted iPSC‐NPCs differentiated into astrocytes in YAC128 TG mice

Finally, we examined whether HLA‐iPSC‐NPCs differentiated into astrocytes in the striatum.

**FIGURE 5:**
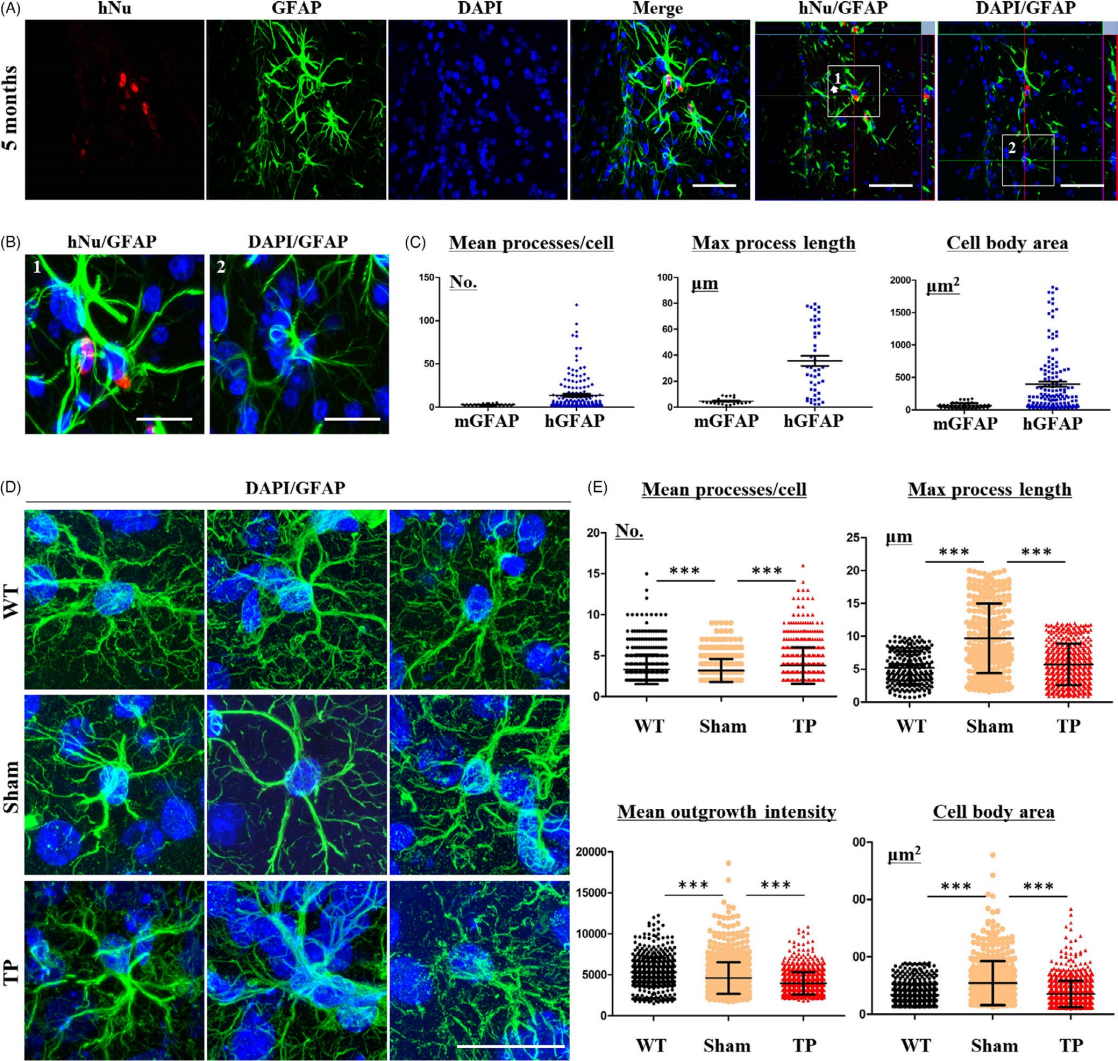
Transplanted iPSC‐NPCs contribute to the differentiation and morphological changes of astrocytes in YAC128 transgenic mice. (A) Double staining for the astrocyte marker GFAP and hNu (Scale bar: 50 μm. 1: merged hNu‐ and GFAP‐positive cells. 2: no merged hNu‐ and GFAP‐positive cells). (B) The difference between hGFAP and mouse GFAP (mGFAP) immunostaining showed morphological changes in HLA‐iPSC‐NPCs‐derived astrocytes. Panel A shows the immunostaining results for hGFAP (1) or mGFAP (2). Scale bar: 20 μm. (C) Quantification of processes, maximal process length, and cell body area in hGFAP‐positive cells and mGFAP‐positive cells (for two regions in 6 brain sections of transplanted group (n = 1)). (D) Morphological differences between endogenous astrocytes in TP mice and WT mice. Scale bar: 20 μm. (E) Quantification of processes, max process length, outgrowth intensity and cell body area in sham, WT and TP mice (for two regions in eight brain sections of each group (n = 3 each), ***P < .001). Data were analysed by two‐way ANOVA followed by a Tukey post hoc test or Student's *t* test using GraphPad's Prism software

Five months after transplantation, we double‐stained hNu‐positive cells with antibodies against GFAP (Figure 5A). To investigate the role of transplanted HLA‐iPSC‐NPC‐derived astrocytes, we compared the human astrocytes from transplanted cells (hNu‐ and GFAP‐positive) with the mouse astrocytes from YAC128 mouse brains (DAPI‐ and GFAP‐positive). The mouse astrocytes were much smaller than the human HLA‐iPSC‐NPCs‐derived astrocytes (Figure 5B). We then analysed the morphology of the HLA‐iPSC‐NPCs‐derived astrocytes using the outgrowth module of MetaXpress software (Molecular Devices). HLA‐iPSC‐NPCs‐derived astrocytes had a higher mean number of processes, maximal process length and body area values than mouse astrocytes (Figure 5C), denoting a more complex structure. Thus, we characterized the morphology of human astrocytes in YAC128 mice.

We next investigated whether the HLA‐iPSC‐NPCs‐derived astrocytes compensated for the dysfunctional astrocytes in the YAC128 transgenic mice. Sham and WT mice had morphologically different astrocytes (Figure 5D). Eleven‐month‐old sham mice had fewer but longer GFAP‐positive processes, indicating reactive astrocytes. The number and intensity of their GFAP‐positive cells were also higher (Figure 5D,E). Importantly, HLA‐iPSC‐NPC transplantation restored the morphology of YAC128 astrocytes. TP mice had a higher mean number of GFAP‐positive processes, but a lower number, intensity and maximal length of GFAP‐positive cells (Figure 5D,E). These results suggest that functional recovery of astrocytes is one of the mechanisms by which the HLA‐iPSC‐NPCs restore neuronal function in YAC128 TG mice.

### 3.6 Transplanted HLA‐iPSC‐NPC‐derived astrocytes showed environmental changes in the YAC128 TG mice

**FIGURE 6:**
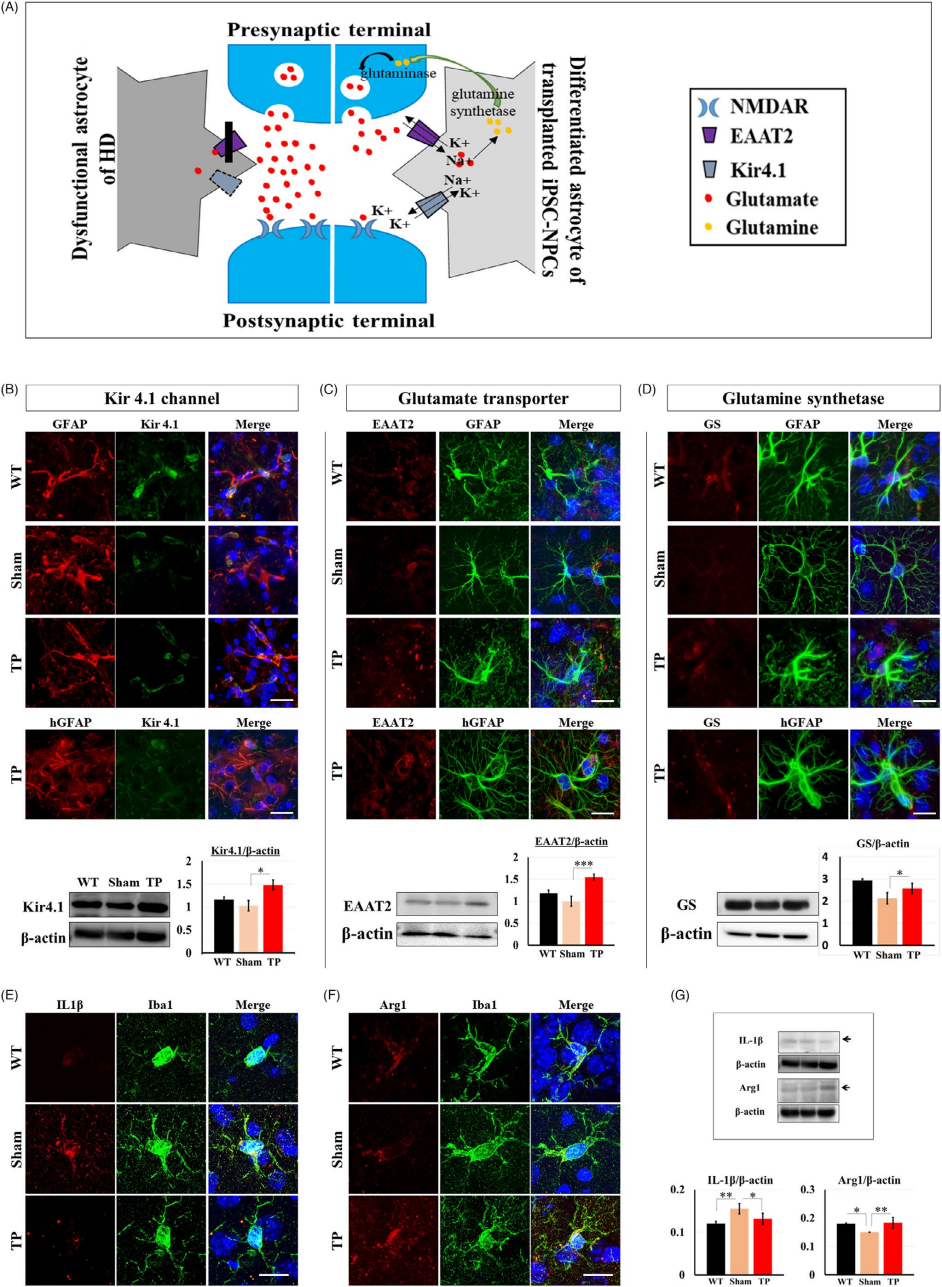
HLA‐iPSC‐NPC‐derived astrocytes modulate environmental toxicity in TP mice. (A) Diagram depicting the glutamate uptake and K+ channel (Kir4.1) buffering in differentiated astrocytes. Differentiated astrocytes of mice with transplanted HLA‐iPSC‐NPCs exhibited recovery of Kir4.1 channel (B), glutamate uptake (C) and glutamine synthetase (GS) (D) for astrocytic functional rescue. Double staining or Western blot analysis for Kir4.1 or EAAT2 or GS and GFAP in 11‐month‐old YAC128 transgenic mice with or without HLA‐iPSC‐NPCs transplants showed that differentiated grafted astrocytes were increased on all three markers compared to the endogenous astrocytes. Scale bar: 20 μm. (B, C and D. n = 3, each group, *P < .05, ***P < .001). (E) Double staining for the inflammatory marker IL‐1β and Iba‐1. Scale bar: 20 μm. (F) Double staining for an anti‐inflammatory marker Arginase1 (Arg1) and Iba‐1. Scale bar: 20μm. (G) Western blot analysis for Arg1 and IL‐1β expression (n = 3 in each group, *P < .05, **P < .01). Data were analysed by two‐way ANOVA followed by a Tukey post hoc test using the SPSS software

To investigate the cellular defects of YAC128 TG astrocytes, we examined the astrocytic Kir4.1 channel current which significantly decreases in the HD brain and HD transgenic animal models.^22^ Astrocytic Kir4.1 channel deficit appears early in the disease process, and the elevated extracellular K+ levels impair spatial K+ buffering, which increases glutamate toxicity in the brain.^23^, ^24^ HLA‐iPSC‐NPCs transplants can potentially modulate several key components of glutamate toxicity (Figure 6A). First, glutamate is transported into astrocytes along with Na^+^ in exchange for K^+^. In HD, striatal astrocytes have a Kir4.1 channel deficit, and thus, the glutamate transporter dysfunction causes functional defects. Second, astrocytes play a crucial role in providing neurons with glutamine, an important precursor for glutamate and GABA synthesis (known as the glutamate‐GABA‐glutamine cycle).

Here, we examined the astrocytes’ abnormal function in YAC128 TG animals and how HLA‐iPSC‐NPCs rescued it. In TP mice, GFAP‐ or hGFAP‐positive cells were Kir4.1‐immunoreactive (Figure 6B). By contrast, GFAP‐positive astrocytes five months after transplantation had a significantly lower Kir4.1 level (Figure 6B). Western blot analysis further confirmed that HLA‐iPSC‐NPCs transplantation restored Kir4.1 expression (Figure 6B). Moreover, TP mice showed higher expression levels of the glutamate transporter EAAT2 in GFAP‐ or hGFAP‐positive cells (Figure 6C). Eleven‐month‐old TP mice had high expression levels of glutamine synthetase, as confirmed by immunostaining and Western blot (Figure 6D).

These results suggest that astrocytes from the transplanted HLA‐iPSC‐NPCs restored the YAC128 TG astrocytes by two complementary mechanisms: rescuing Kir4.1 expression (thus increasing glutamate uptake) and increasing glutamine synthetase expression (thus increasing glutamine production).

Astrocytes play anti‐inflammatory roles by releasing various factors to the microglia.^25^ Therefore, we investigated whether astrocytes differentiated from the transplanted HLA‐iPSC‐NPCs could influence microglia polarization, thereby reducing inflammation. We found immunohistochemical evidence that TP mice had significantly lower IL1β levels (a pro‐inflammation marker) and significantly higher Arginase1 (Arg1, an anti‐inflammatory marker) levels than sham mice (Figure 6E,F). Western blot analysis confirmed this result (Figure 6G). Results from the additional pixel analysis (Figure S4) to quantify the intensity of each fluorescence images shown in Figure 6B‐F were consistent with Western blot analysis.

In HD, expression of brain‐derived neurotrophic factor (BDNF) is significantly reduced. In immunohistochemistry, we observed that hGFAP‐positive cells were co‐localized with BDNF‐positive cells in TP group (Figure S5A). Besides, TP mice had higher expression levels of tropomyosin receptor kinase B (TrkB, a BDNF receptor) than sham mice (Figure S5B). Altogether, these results strongly suggest that HLA‐iPSC‐NPCs transplantation changed the environmental neurotoxicity of the mouse or human astrocytes.

## 4 DISCUSSION

This study demonstrates the multipotential differentiation of transplanted HLA‐iPSC‐NPCs and the neuroprotective effects of each differentiated cell type in YAC128 HD transgenic mice. Our results suggest that modulating environmental toxicity through HLA‐iPSC‐NPC transplantation could be a useful therapeutic strategy against HD.

The effect of neural transplantation against HD has been assessed with foetal striatal tissues, neural stem cells (of both foetal and embryonic stem cell origin) and isolated astrocyte‐biased glial progenitor cells. While immunodeficient mice have allowed assessment of donor cell behaviour in the absence of adaptive inflammatory responses, human allografts will require immune suppression. Indeed, immune rejection has been observed in human allografts, despite the use of immunosuppressive agents,^26^, ^27^ possibly reflecting the difficulty in maintaining long‐term compliant immunosuppression. Therefore, we decided to assess the use of HLA‐homozygous iPSC lines that we recently established.^11^ They should reduce the need for immunosuppressive agents when transplanted into HLA‐matched patients. Although more rigorous tests will be necessary using humanized HD animal models in the future, this approach is currently under assessment in initial clinical trials of cell therapy in both age‐related macular degeneration^28^ and Parkinson's disease patients.^29^

Thus, we used HLA‐iPSC‐NPCs and found that the transplanted cells survived and differentiated into the three major neural lineages (neurons, astrocytes and oligodendrocytes), as previously reported.^30^, ^31^

YAC128 transgenic mice express the full‐length mutant huntingtin protein and replicate the motor dysfunction and neuropathology observed in human HD.^12^ Behaviourally, two‐month‐old mice display early motor functions deficits on the rotarod and grip strength tests,^32^ whereas cognitive dysfunctions revealed by the simple swim test and swimming T‐maze test appear later at nine months of age.^9^ In this study, we showed that transplanted HLA‐iPSC‐NPCs into the YAC128 transgenic mouse model of HD differentially differentiated into the three major neural lineages, leading to functional recovery.

First, transplanted HLA‐iPSC‐NPC‐derived neurons have neuroprotective effects. In eight‐month‐old YAC128 transgenic mice, neuronal atrophy decreases the striatal volume, while neuronal loss becomes evident by 12 months of age. However, in the striatum, the expression of DARPP‐32 and synaptophysin start decreasing from six months of age.^33^, ^34^ We showed that HLA‐iPSC‐NPCs transplantation increased the expression of DARPP‐32‐ and synaptophysin. Their neuronal differentiation in YAC128 mice may increase the expression of DARPP‐32 and synaptophysin in the striatum.

Next, transplanted HLA‐iPSC‐NPC‐derived oligodendrocytes helped myelination in the CC. MSN degeneration in the caudate and putamen regions of the basal ganglia has long been considered the major neuropathological hallmark of HD. Myelin breakdown and white matter atrophy also appear to be universal features of the disease.^35^, ^36^ Electron microscopy analysis of myelinated fibres of the CC indicated that myelin sheaths are thinner in YAC128 mice as young as 1.5 months, well before any neuronal loss can be detected.^34^ Here, HLA‐iPSC‐NPCs transplantation increased the thickness and density of MBP in YAC128 mice. This may be due to the differentiated oligodendrocyte near the CC.

Finally, mouse astrocytes or transplanted HLA‐iPSC‐NPC‐derived astrocytes decreased environmental toxicity in YAC128 transgenic mice. Human astrocytes are larger and have more processes than mouse astrocytes.^37^, ^38^, ^39^ The HLA‐iPSC‐NPC‐derived astrocytes were larger and had more processes than mouse astrocytes. Besides, mouse astrocytes in TP mice shared more morphological features with astrocytes from WT mice than with those from sham mice. In TP mice, we observed the respective functions of mouse astrocytes or human astrocytes. The present study focused on two aspects of astrocytic reduction in the environmental toxicity: (1) glutamate buffering and (2) anti‐inflammatory actions.

### 4.1 Glutamate toxicity control

Astrocytes remove the spillover of synaptic neurotransmitters such as glutamate. In HD, the expression of the two glia‐specific transporters which accomplish this, EAAT1 and EAAT2 38, 39, 40, is low. Moreover, there is a loss of the astrocytic Kir4.1 channel in HD, which impairs K+ buffering and further increases glutamate toxicity.^22^, ^23^, ^24^ TP mice displayed no loss of the astrocytic Kir4.1 channel and an increased expression of EAAT2.

### 4.2 Rescue of the glutamate‐GABA‐glutamine cycle

In the brain, astrocytes are the most abundant cell type and are tightly associated with synapses, since they synaptic transmission and provide metabolic support to neurons. In HD, astrocyte metabolism is impaired. In particular, the glutamate‐GABA‐glutamine cycle dysfunction reduces GABA production.^40^, ^41^ Thus, normalizing the impaired astrocyte metabolism could be beneficial to HD patients. We found that HLA‐iPSC‐NPCs transplantation increased glutamine synthase expression by astrocytes, implying a recovery of impaired astrocyte metabolism.

### 4.3 Anti‐inflammatory action

Astrocytes can also act as secretory cells by producing neurotransmitters, neuromodulators, neurohormones, cytokines, neurotrophic factors, etc Released cytokines and neurotrophic factors play anti‐inflammatory roles in various neurodegenerative diseases.^25^ For example, increasing the production of BDNF by astrocytes reduced inflammation in transgenic HD mice.^42^ Also, astrocytes release anti‐inflammatory factors such as TGF‐β, and HD astrocytes have low TGF‐β mRNA and protein levels.^43^ TGF‐β release by astrocytes can decrease inflammation. Therefore, astrocyte differentiation by transplanted HLA‐iPSC‐NPCs could restore anti‐inflammatory action. In line with this, we found that HLA‐iPSC‐NPC‐derived astrocytes expressed BDNF and TrkB. Altogether, the normal function of differentiated astrocytes can improve the inflammatory environment of the HD brain.

In conclusion, transplanted HLA‐iPSC‐NPCs differentiate into the three major neural lineages (neurons, oligodendrocytes and astrocytes) and can exert neuroprotective effects in the HD YAC128 transgenic mouse model. Using HLA‐homozygous cells, we aim to develop an immune‐compatible allogeneic transplantation approach. Here, we showed that HLA‐iPSC‐NPC transplantation helped to recover several impaired functions. This approach could suppress immune rejection in allogeneic clinical settings, which would be promising for the future treatment of HD patients.

## 5 DECLARATION OF INTERESTS

JS is the founder and CEO of iPS Bio, Inc The other authors declare that they have no competing interests.

## AUTHOR CONTRIBUTIONS

HJP designed the study, performed the majority of experiments, analysed the data, wrote the manuscript and provided financial support; JJ prepared HLA‐iPSC‐NPCs for transplantation experiment; JC and JYK helped with the animal experiments and discussed the results; HSK, JYH and SAG discussed and interpreted the results; JS supervised the entire study, provided financial support, wrote the manuscript and approved the final manuscript.

## Supporting information

Fig S1‐5

Video S1

Video S2

Video S3

Video S4

## Data Availability

The data that support the findings of this study are available from the corresponding author upon reasonable request.
