# Supplementary material for: Human iPSC‐derived neural precursor cells differentiate into multiple cell types to delay disease progression following transplantation into YAC128 Huntington's disease mouse model
Source: Cell Prolif. 2021 Jun 21;54(8):e13082. doi: 10.1111/cpr.13082 (PMC8349664; doi:10.1111/cpr.13082)
Supplement: Supplementary file 1 — Fig S1‐5 [file CPR-54-e13082-s003.docx]

**Supplemental Information**

**Human iPSC-derived neural precursor cells differentiate into multiple cell types to delay disease progression following transplantation into YAC128 Huntington’s disease mouse model**

Hyun Jung Park,^1^ Juhyun Jeon,^1^ Jiwoo Choi,^1^ Ji Yeon Kim,^1^ Hyun Sook Kim,^2^ Ji Young Huh^3^, Steven A. Goldman,^4,5^ and Jihwan Song^1,6,*^

^1^Department of Biomedical Science, CHA Stem Cell Institute, CHA University, 335 Pangyo-ro, Bundang-gu, Seongnam-si, Gyeonggi-do 13488, Korea

^2^Department of Neurology, CHA Bundang Medical Center, CHA University, 59 Yatap-ro, Bundang-gu, Seongnam-si, Gyeonggi-do 13496, Korea

^3^Department of Laboratory Medicine, CHA Bundang Medical Center, CHA University, 59 Yatap-ro, Bundang-gu, Seongnam-si, Gyeonggi-do 13496, Korea

^4^Center for Translational Neuromedicine, University of Rochester Medical Center, Rochester, NY 10021, USA

^5^Center for Translational Neuromedicine, University of Copenhagen Faculty of Health and Medical Science, 2200 Copenhagen N, Denmark

^6^iPS Bio, Inc., 3F, 16 Daewangpangyo-ro 712 Beon-gil, Bundang-gu, Seongnam-si, Gyeonggi-do 13522, Korea

*Correspondence:

Jihwan Song, D.Phil.

CHA Stem Cell Institute, Department of Biomedical Science, CHA University, Rm 604, CHA Bio Complex, 335 Pangyo-ro, Bundang-gu, Seongnam-si, Gyeonggi-do 13488, Republic of Korea
E-mail: [jsong5873@gmail.com](mailto:jsong@cha.ac.kr)

**Supplemental Information contains:**

**5 Supplemental Figures**

**Figure S1.** HLA-iPSC-NPCs attenuate survival in 11 months-old YAC128 transgenic mice. Related to Figure 2.

**Figure S2.** The reduction of rotarod test has not significant difference from 3 months to 5 months after transplantation. Related to Figure 2.

**Figure S3.** Quantification of differentiated neurons, oligodendrocytes or astrocytes in YAC128 mice with HLA-iPSC-NPCs transplant. Related to Figure 3, 4, and 5.

**Figure S4.** Pixel analysis of each immunofluorescence images in Figure 6B-F. Related to Figure 6.

**Figure S5.** HLA-iPSC-NPCs attenuate survival in 11-month-old YAC128 transgenic mice. Related to Figure 6.

**4 Supplemental Videos**

**Video S1.** Motor function – rotarod test. Related to Figure 2.

**Video S2.** Motor function – grip strength test. Related to Figure 2.

**Video S3.** Cognitive function – simple swim test. Related to Figure 2.

**Video S4.** Cognitive function – novel object recognition test. Related to Figure 2.

**
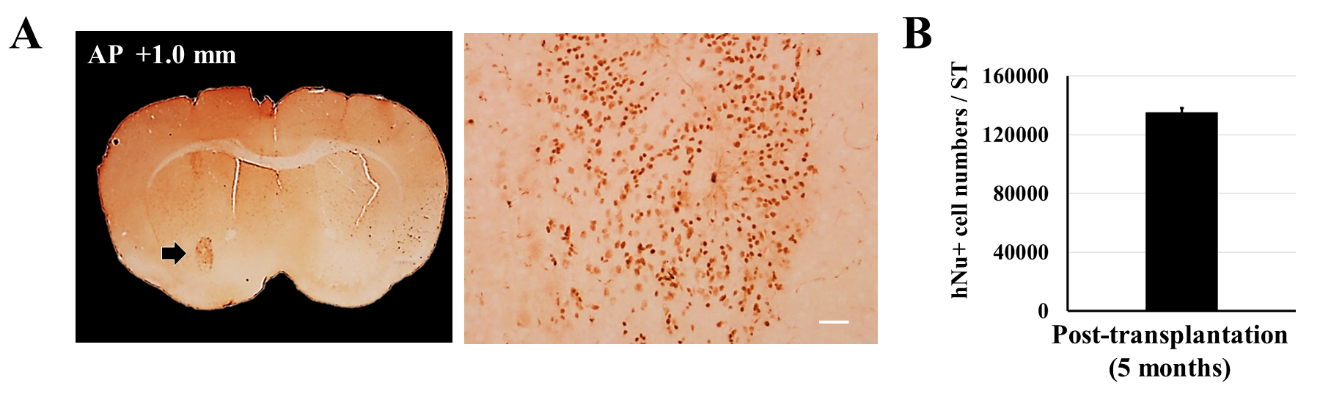
**

**Figure S1. Survival and detection of HLA-iPSC-NPCs in 11 month-old YAC128 transgenic mice**

(A) Immunostaining for the identification of human nuclei (hNu)-positive cells, indicating the survival of transplanted humans cells in the striatum. Scale bar: 200 μm. (B) Quantification of hNu-positive cell numbers in the whole striatum (n = 5).

**
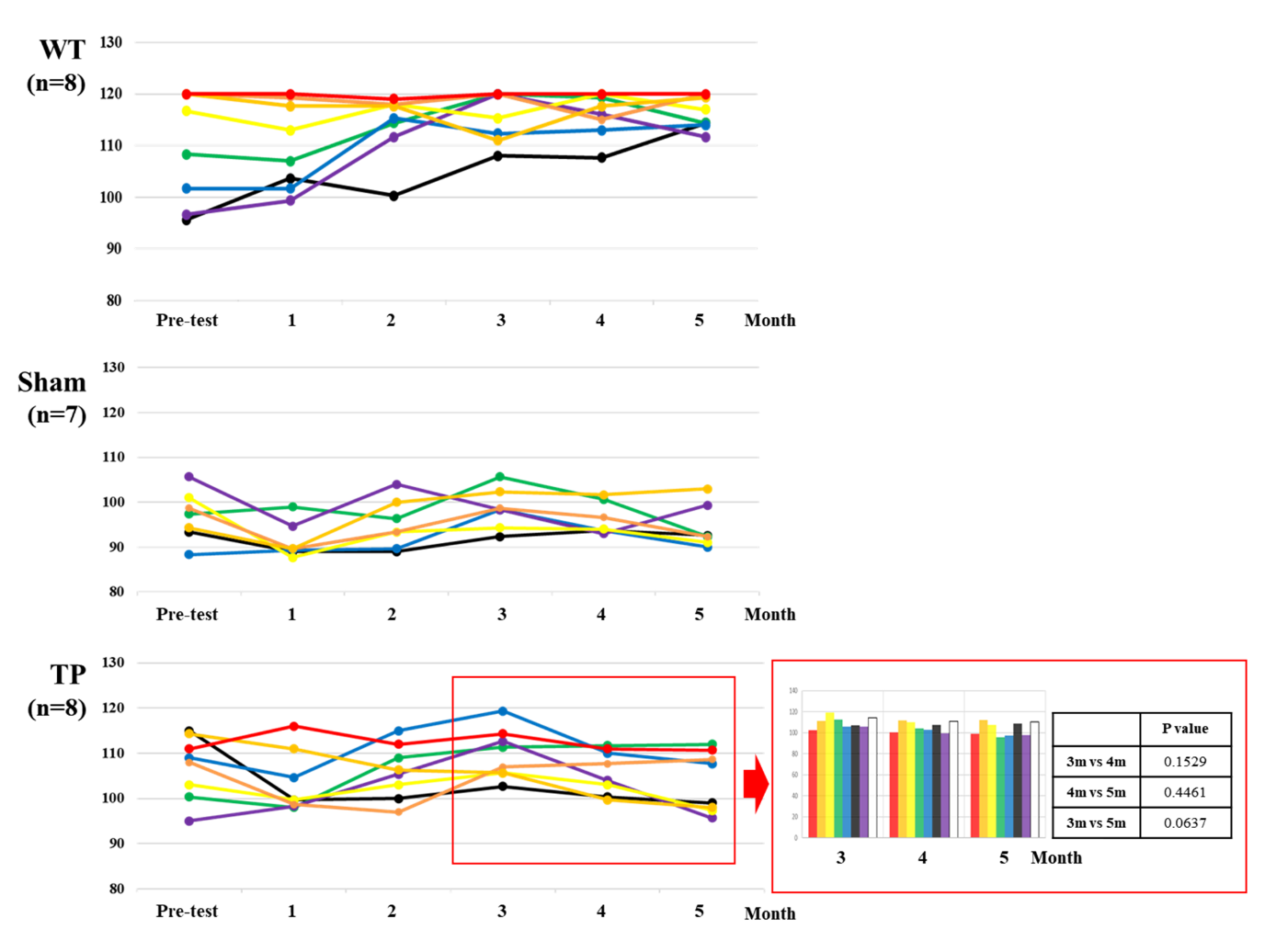
**

**Figure S2. Results from the rotarod test showing no significant changes of scores from three to five months following transplantation**

In the transplanted group (n=8), no statistical differences were observed after three months in the following comparison groups: 3 months vs. 4 months; 4 months vs. 5 month; 3 months vs. 5 months

**
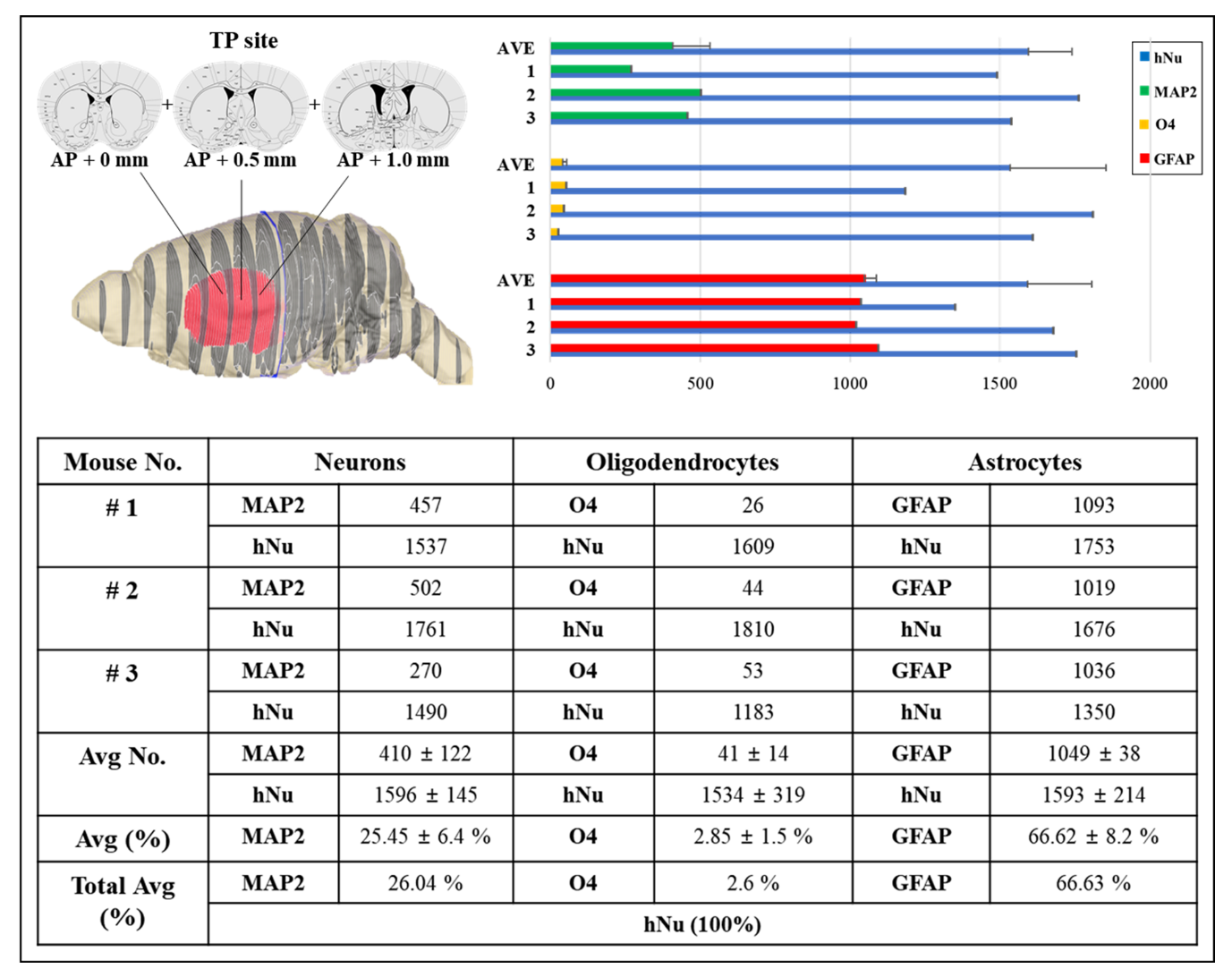
**

**Figure S3. Quantification of the proportions of neurons, oligodendrocytes and astrocytes in YAC128 mice transplanted with HLA-iPSC-NPCs.**

The percentages of merged MAP2-, O4-, or GFAP- positive cells against human nuclei antigen (hNu) were analyzed in the striatum of three brain sections (AP=0, 0.5, 1.0 mm, n=3) using the IXMC high-content imaging system. The percentages of double-positive cells against hNu in each analysis were shown.

**
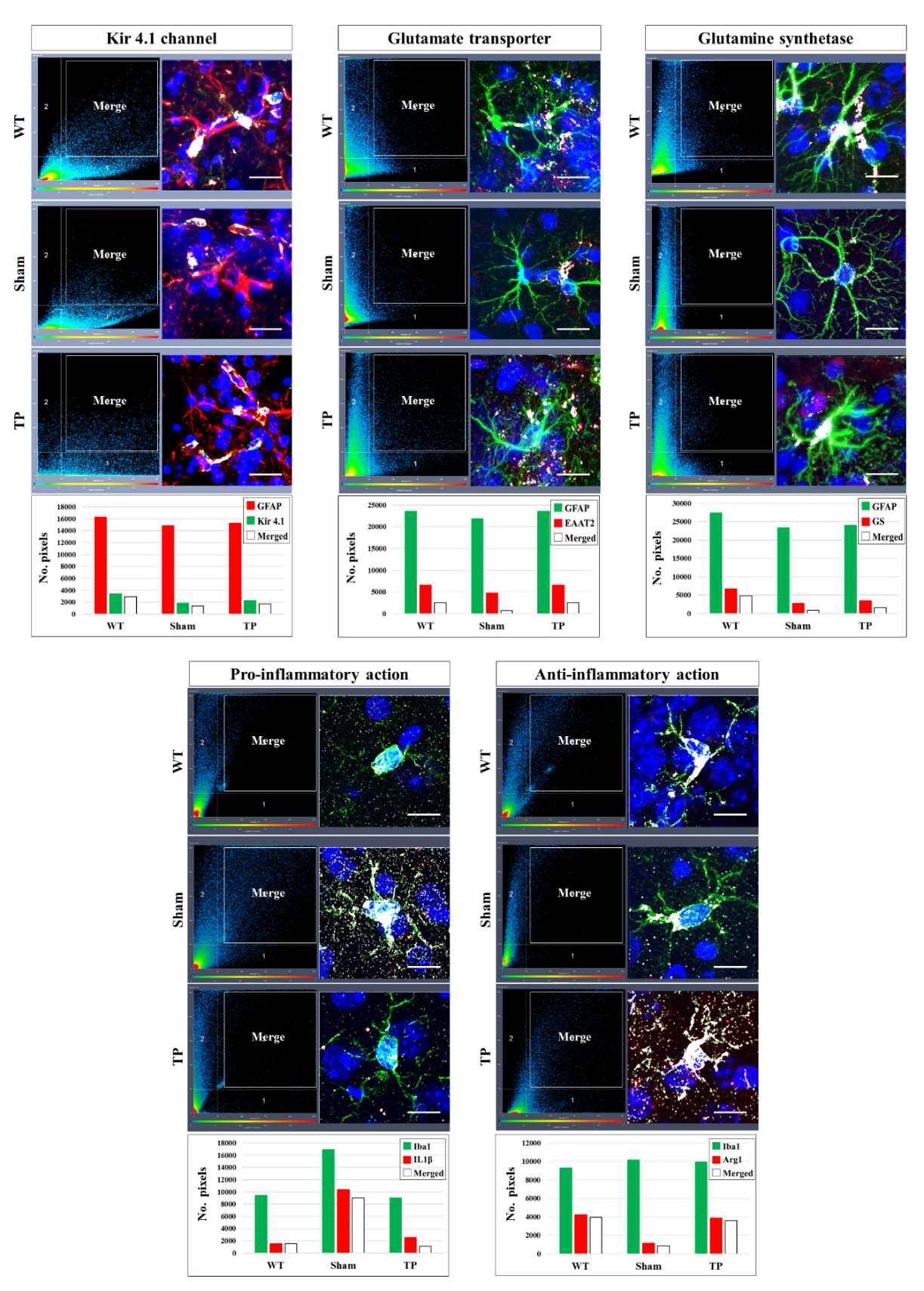
**

**Figure S4. Pixel analysis to quantify the intensity of fluorescence signals.** Each fluorescence image shown in Figure 6B-F were measured using the co-localization module of Zen black software of LSM9 confocal microscope (Zeiss). The intensity of each fluorescence image was quantified.

**
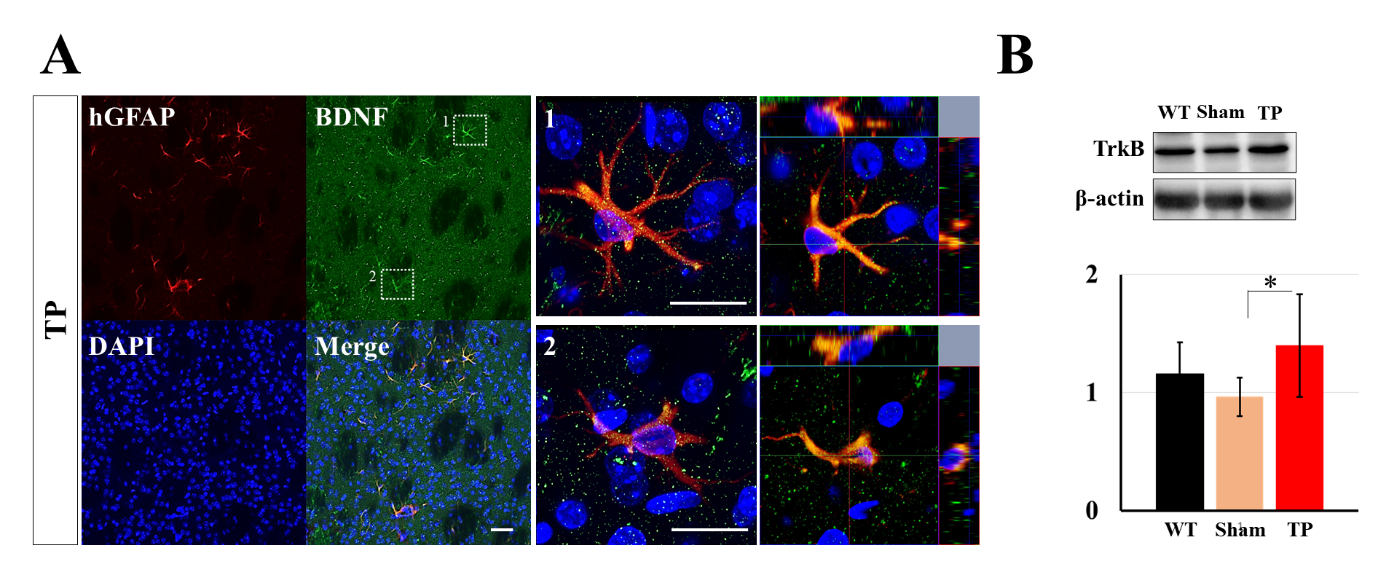
**

**Figure S5. Expression of BDNF-positive cells in 11 month-old YAC128 transgenic mice**

(A) Double staining for hGFAP and brain-derived neurotrophic factor (BDNF). 1 and 2 are high magnifications of two representative results from the transplanted group. Scale bar: 20 μm. (B) Western blot analysis on the expression of tropomyosin receptor kinase B (TrkB) in the transplanted mice (n = 3 in each group, *p < 0.05).
